# *Typhlonesticus gocmeni* sp. n., a new cave-dwelling blind spider species from the Aegean region of Turkey (Araneae, Nesticidae)

**DOI:** 10.3897/zookeys.419.5739

**Published:** 2014-06-23

**Authors:** Carles Ribera, Mert Elverici, Kadir Boğaç Kunt, Recep Sulhi Özkütük

**Affiliations:** 1 Institut de Recerca de la Biodiversitat, Departament de Biologia Animal. Universitat de Barcelona, Av. Diagonal, 643, Barcelona-08028, Spain; 2 Department of Biology, Faculty of Science and Arts, University of Erzincan, TR-24100, Erzincan, Turkey – Department of Biological Sciences, Faculty of Arts and Sciences, Middle East Technical University, TR-06800 Ankara, Turkey; 3 Poligon Sitesi 71/27-B TR-06810 Dodurga, Çayyolu, Ankara, Turkey; 4 Department of Biology, Faculty of Science, Anadolu University, TR- 26470 Eskişehir, Turkey

**Keywords:** Arachnida, taxonomy, description, caves, Anatolia, troglobiont

## Abstract

A new species of the troglobitic spider genus *Typhlonesticus* is described from specimens found in Keloğlan Cave (Denizli Province, Dodurgalar Town), Turkey. *Typhlonesticus gocmeni* **sp. n.** is described on the basis of both sexes; and its phylogenetic relationships with closely related European genera and species are discussed based on morphological and molecular data (the *cox1*, *rrnL* and *H3* genes). Three new combinations are proposed: *Typhlonesticus idriacus* (Roewer, 1931), **comb. n.**, *Typhlonesticus morisii* (Brignoli, 1975) **comb. n.** and *Typhlonesticus obcaecatus* (Simon, 1907), **comb. n.** all ex *Nesticus*.

## Introduction

Nesticids are medium-sized spiders common in underground ecosystems in the northern Mediterranean basin and many of them exhibit all the typical troglobite characters: depigmentation, anophthalmia and appendage lengthening. The following four genera have been recorded from this region: *Aituaria* Esyunin & Efimik, 1998; *Carpathonesticus* Lehtinen & Saaristo, 1980; *Nesticus* Thorell, 1869 and *Typhlonesticus* Kulczyński, 1914, representing a total of 49 species. Two more genera occur in bordering regions: *Canarionesticus* Wunderlich, 1992, endemic to the Canarian archipelago and *Nesticella* Lehtinen & Saaristo, 1980, broadly spread throughout the Asian continent. *Nesticella mogera* (Yaginuma, 1972) was recorded from the southeast of Caucasus (Marusik and Guseinov 2003), and in Europe has been cited in Berlin and Poland (Bielak-Bielecki and Rozwałka 2011) as an introduced species.

Most described species are well defined and illustrated, but the taxonomy of the group is not well established at the genus level, mainly in *Carpathonesticus* and *Nesticus* which show conspicous morphological variability in their genital organs, suggesting the existence of independent evolutionary lineages. This morphological variability was already pointed out by Lehtinen and Saaristo (1980) and López-Pancorbo and Ribera (2011).

The genus *Typhlonesticus* was described in 1914 and included two species, but a type species was not selected. Kratochvíl (1933) designated the type species, but incorrectly. His designation was corrected by Lehtinen and Saaristo (1980). Currently the genus comprises the single species *Typhlonesticus absoloni* (Kratochvíl, 1933), a species known from several caves in Montenegro (Platnick 2013). The phylogenetic affinities of this genus are unknown and it has been regarded by some authors as an aberrant species, “*the most aberrant of Nesticini in regard to both female and male genital organs is Typhlonesticus, and its relationships to other genera remain obscure*” (Lehtinen and Saaristo 1980) or “*an old relict,... with a solitary position within the genus*” (Deeleman-Reinhold 1974). However, Fage (1931) reported some morphological relationships with regard to *Nesticus obcaecatus* (*la forme de l’epigyne rappelle un peu celle du N. obcaecatus de l’Espagne*) and López-Pancorbo and Ribera (2011) pointed out the morphological affinities of the male and female copulatory organs between *Typhlonesticus absoloni* and *Nesticus obcaecatus*, *Nesticus idriacus* and *Nesticus morisii*.

An extensive survey of caves in Anatolia during the last 10 years has provided a high number of morphospecies, one of which, from Keloğlan Cave in the Denizli Province, shows a clear morphological similarity with *Typhlonesticus absoloni*. The discovery of this new species of *Typhlonesticus* has also led us to review some of the described Mediterranean species that show morphological similarity with *Typhlonesticus absoloni*, such as *Nesticus obcaecatus* from the Spanish Pyrenees, *Nesticus morisii* from Italy and *Nesticus idriacus* from Austria and Italy. In order to check the phylogenetic relationships among these species a molecular phylogenetic analysis based on nuclear and mitochondrial gene sequences was performed.

The aim of this paper is to describe a new species belonging to the genus *Typhlonesticus* and to propose three new combinations of the above mentioned species.

## Material and methods

### Molecular data

**Taxonomic sampling.** Representatives of Mediterranean *Nesticus*, *Carpathonesticus* and *Typhlonesticus* were included in the analysis. We could not include representatives of the genus *Aituaria*, the easternmost Mediterranean nesticid genus whose range extends from southern Urals to the northwest of Caucasus, due to lack of suitable material for molecular analysis. Sequences from *Canarionesticus quadridentatus* Wunderlich, 1992 from Canary Islands were used to root the tree. We have also included sequences of *Typhlonesticus absoloni*, *Nesticus idriacus*, *Nesticus morisii* and *Nesticus obcaecatus* Simon, to check the phylogenetic relationships of these species (see Appendix 1 for localities and GenBank accession numbers).

**Sample storage and DNA extraction.** For DNA studies, live specimens were collected in the field, fixed in 96% or absolute ethanol and stored at 4 °C. Total genomic DNA was extracted from legs or from the prosoma of a single specimen using the QIamp® DNA Mini Kit (QIAGEN) following the manufacturer’s protocols. The approximate concentration and purity of the DNA obtained were verified using 1% agarose/TBE gel electrophoresis.

**PCR amplification and sequencing.** Partial fragments of two mitochondrial (cytochrome oxidase I: *cox1* and 16S rRNA: *rrnL*) and one nuclear (Histone 3: *H3*) genes were selectively amplified and sequenced using the primers and conditions shown in Appendix 2. The PCR reaction mixture contained a final concentration of 0.2 μM of each primer, 0.2 mM of each dNTPs, 0.5 U Taq polymerase (Promega), with the supplied buffer, and 1.5–2.5 mM MgCl_2_ in a final volume of 25 μL. The PCR products were cycle-sequenced in both directions using the same PCR primers and the BigDye Terminator version 3.1 Cycle Sequencing Kit (Applied Biosystems) and analyzed using an ABI 3700 automated sequencer at the Serveis Científico-Tècnics of the Universitat de Barcelona.

**Alignment**, **genetic distances and phylogenetic analyses.** Raw sequences were edited and assembled with GENEIOUS v4.6.5 (Drummond et al. 2009). The new sequences used in this study have been deposited in GenBank under the accession numbers shown in Appendix 1. Alignment of the *cox1* and *H3* gene fragments was trivial due to the absence of length polymorphism. However, there were some length differences among the *rrnL* fragments, suggesting the occurrence of insertion/deletion events during the evolution of these sequences. Automatic alignment algorithms have been considered as superior to manual protocols due to their objectivity and repeatability (Giribet et al. 2002). The *rrnL* sequences were aligned using the online version of MAFFT, applying the Q-INS-i algorithm (Katoh and Toh 2008). The uncorrected genetic distances between the taxa were assessed using MEGA v5.0 (Tamura et al. 2011).

Maximum Likelihood (ML) analyses of the combined data matrix corresponding to the three sequenced genes were conducted using the online version of RAxML (Stamatakis et al. 2008), independently applying the parameters of the Gamma model of rate heterogeneity for each partition. The online version obtains the bootstrap support values by means of 100 pseudoreplicates.

### Taxonomy

The following abbreviations are used in the text and figures: E = embolus, T = tegulum, ST = subtegulum, MA = median apophysis, TTA = theridioid tegular apophysis, p1 = process 1 of TTA, p2 = process 2 of TTA, P = paracymbium, vp = ventral process of paracymbium, dp = dorsal process of paracymbium, co = copulatory orifice, id = insemination duct, fd = fertilization duct, S = spermatheca, AUZM Anadolu University Zoological Museum (Eskişehir, Turkey), CRBA Centre de Recursos de Biodiversitat Animal de la Universitat de Barcelona (Spain). All measurements are in millimetres.

Specimens of *Typhlonesticus gocmeni* sp. n. were collected using hand aspirators and placed directly into 96% ethanol in the field. Body colour descriptions are based on digital images taken in the cave environment. Photography was performed using a Nikon D100 camera equipped with a Nikon 105mm f/2.8G ED-IF AF-S VR Micro-Nikkor lens and a Sigma EM-140 DG macro ring flash for Nikon SLR cameras.

The female vulva was removed and treated with 30% KOH prior to examination. After observation and drawings, the vulva was washed in distilled water and stored in 70% ethanol. The left male palp was drawn in all cases. We follow Coyle and McGarity (1992) for describing the paracymbium and Huber (1993) and Agnarsson et al. (2007) for other parts of the male and female copulatory organs.

Digital images of the palps and vulvae were taken with a Leica DFC295 digital camera attached to a Leica S8AP0 stereomicroscope, with 5–15 photographs taken in different focal planes and combined using image stacking software. Photographic images were edited using PHOTOSHOP CS2 and COREL-DRAW X3 was used to create the plates. For SEM micrographs, the male palps were dried at -30 °C and coated with a thin layer of gold using an Electron Microscopy sciences EMS 550X sputter coater. The materials were examined at an acceleration voltage of 12 kV using a ZEISS ULTRA PLUS Scanning Electron Microscope (University of Anadolu, Eskişehir, Turkey).

In addition of the new species we also examined the following material: *Nesticus morisii* (♀) from Sotterranei del Forte di Vernante, Vernante, Cuneo, Italy, 19.09.2007, leg. A. López-Pancorbo & M. Isaia; *Nesticus idriacus* (♂♀) from Grotte Pre Oreak, Nimis, Friuli. Italy, 15.09.2007, leg. A. López-Pancorbo; *Nesticus obcaecatus* (♂♀) from Cueva Del Molino de Aso, Boltaña, Prov. Huesca, Spain, 27.05.2004, leg. S. Carranza; and *Typhlonesticus absoloni* (♀) from Baba Tusha Cave, Trnovo, Virpazar Distr., Montenegro, 24.03.2006, leg. B. Petrov & S. Lazarov. Baba Tusha Cave is located about 10 km in a straight line from Grboćica pećina, in Krivośije (locus typicus of *Typhlonesticus absoloni*) and about 20 km away from Cetinsjska pećina, from which Deeleman-Reinhold (1974) illustrated both sexes of this species. For morphology of the male palps of *Typhlonesticus absoloni* and *Nesticus morisii* we rely on Kratochvíl (1939) and Deeleman-Reinhold (1974).

## Results

### Taxonomy
Family Nesticidae Simon, 1894
Genus *Typhlonesticus* Kulczyński, 1914

**Type species.** *Nesticus absoloni* Kratochvíl, 1933; see Lehtinen and Saaristo (1980).

#### Typhlonesticus gocmeni sp. n.

Taxon classificationAnimaliaAraneaeNesticidae

http://zoobank.org/E3A3721A-0E71-479B-9EA9-A31A47D6E8C6

Figs 2 [Fig F3] [Fig F4] [Fig F5] –21

##### Material examined.

***Holotype*** ♂ (AUZM) Denizli Province, Acıpayam District, Dodurgalar Town, Keloğlan Cave (37°23'14.74"N, 29°34'18.29"E), 10.07.2011, leg. M. Elverici. ***Paratypes*** 1 ♂ 1 ♀ 9 juveniles (AUZM), 1 ♂ 1 ♀ (CRBA) same data as holotype.

##### Derivatio nominis.

The specific name is given in honour of the prominent Turkish biologist, Prof. Dr. Bayram Göçmen (University of Ege, İzmir, Turkey). Noun in apposition.

##### Diagnosis.

Males of the new species differ from *Typhlonesticus absoloni* by the shape and length of the tegulum, the shape of p1 and p2 processes, the arrangement of the embolus and the shape and arrangement of the paracymbial apophyses, mainly the ventral one, which is erected as a thin spine in *Typhlonesticus gocmeni* sp. n., whereas in *Typhlonesticus absoloni* it consists of a curved lamella. Females differ from *Typhlonesticus absoloni* by the shape of the epigyne and the position of the spermathecae. The dimension and orientation of the insemination and fertilization ducts are also diagnostic. In *Typhlonesticus gocmeni* sp. n. the epigyne is scarcely sclerotized and the spermathecae and insemination ducts are visible through the tegument, moreover the spermathecae are nearly spherical and separated by a distance approximately equal to their diameter. In *Typhlonesticus absoloni* the epigyne is strongly sclerotized and the spermathecae are separated by almost twice their diameter. In *Typhlonesticus absoloni* the insemination and fertilization ducts are thicker and almost fill the entire genital area.

##### Male.

***Coloration*.** Carapace whitish, slightly yellowish. Appendages and sternum slightly testaceous. Opisthosoma brownish, with many dark patches (Fig. 20). The specimens preserved in alcohol have a whitish opisthosoma, slightly greyer than the prosoma.

***Prosoma*.** Carapace approximately circular in dorsal view. Cephalic region not differentiated from the rest of the carapace. Eyeless (Figs 18, 19).

***Opisthosoma*.** Sub-elliptical in dorsal view.

***Appendages*.** prolateral margin of the chelicerae with 3 teeth, the central slightly longer. ***Male palp*** (Figs 2–13). Paracymbium short, dorsal and ventral processes scarcely developed. The ventral process consists of a short and flattened lamella, curved towards the apex and prolonged into a thin spine. The ventral one consisting of a short laminar apophysis, apically curved toward to the ventral side. Distal, paradistal and dorsomedian paracymbial apophyses absent (Figs 2–3 and 5–7). Tegulum very prominent, consisting of a ventrally directed triangular apophysis. Small inconspicuous median apophysis located behind the tegulum (Figs 2, 4, 6, 8, 12). TTA with two well developed processes (p1 and p2): p1 is saddle-shaped, longer than wide, slightly curved in the middle and directed ventrally; p2 is in an apical position and ends with two convergent apical hooks running as a conductor for the embolus (Figs 2, 4–6, 8, 11, 13). Embolus filamentous following a semicircular course towards the apex and bordering the tegulum.

**Figure 1.**
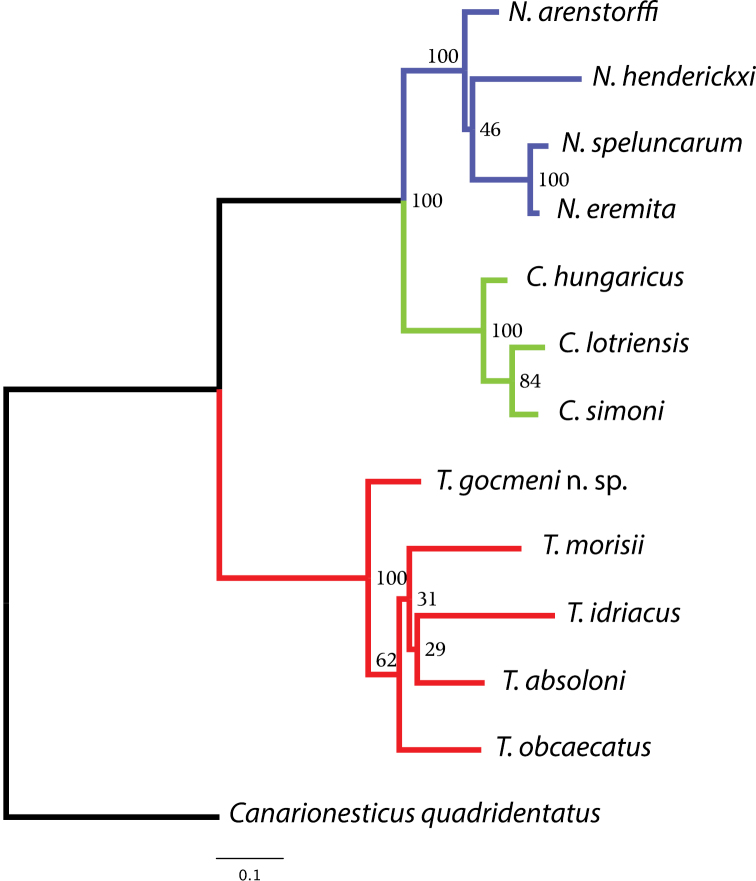
ML tree inferred using the concatenated dataset of *cox1*, *rrnL* mtDNA and *H3* nuDNA gene fragments. Numbers next to nodes correspond to bootstrap support values. The tree was rooted using *Canarionesticus quadridentatus* from the Canary Islands.

**Figures 2–5.**
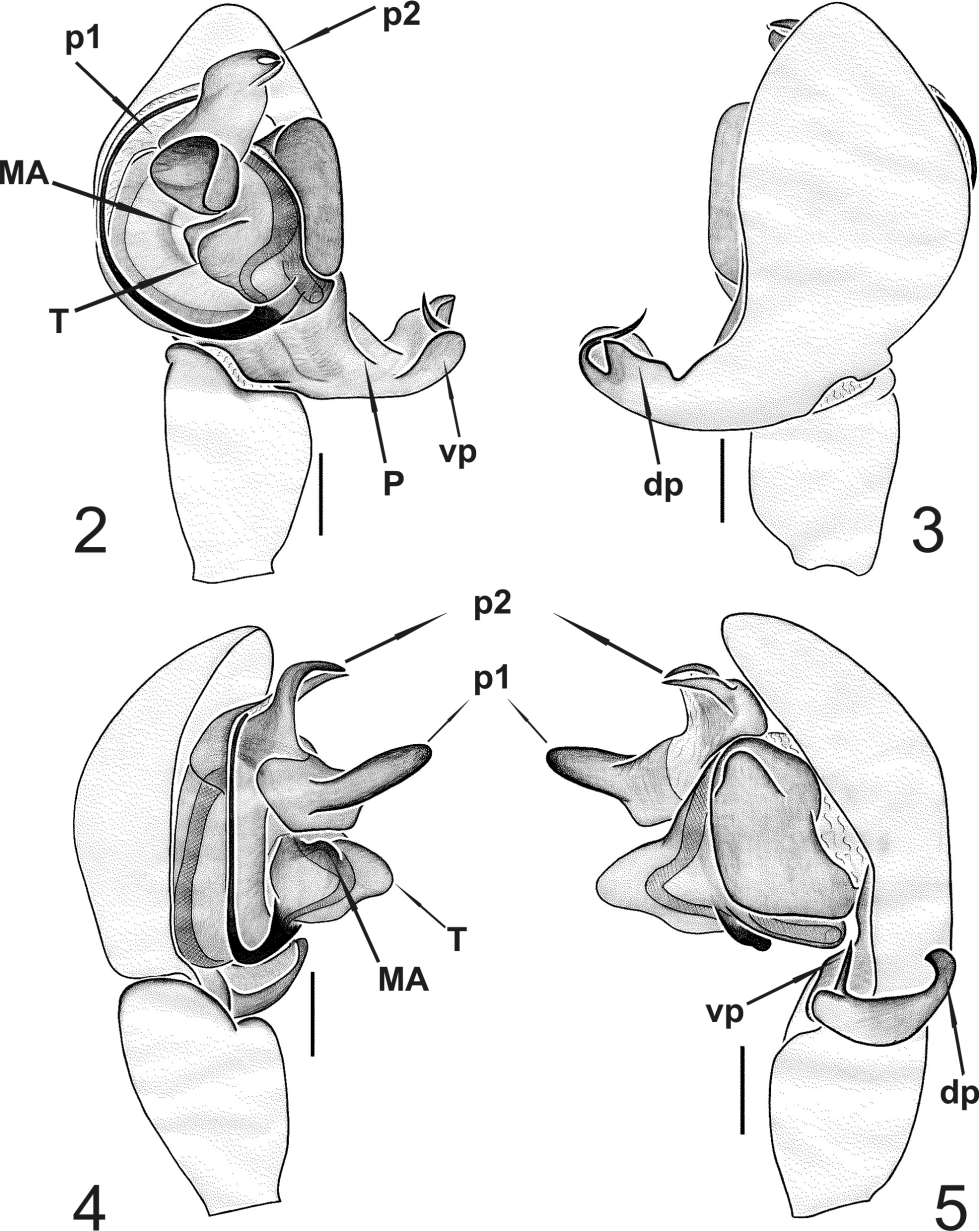
*Typhlonesticus gocmeni* sp. n. male palp. **2** ventral view **3** dorsal view **4** prolateral view **5** retrolateral view. Abbreviations: **T** = tegulum, **MA** = median apophysis, **p1** = process 1 of TTA, **p2** = process 2 of TTA, **P** = paracymbium, **vp** = ventral process of paracymbium, **dp** = dorsal process of paracymbium. Scale bars 0.1 mm.

**Figures 6–9. F3:**
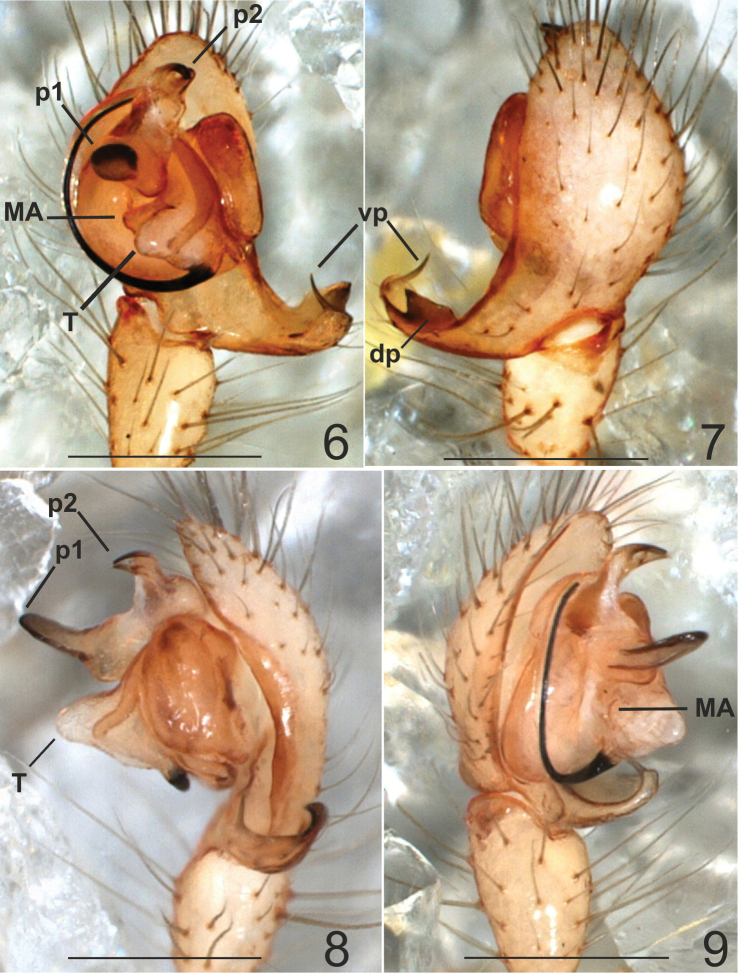
*Typhlonesticus gocmeni* sp. n. male palp. **6** ventral view **7** dorsal view **8** retrolateral view **9** prolateral view. Abbreviations: **T** = tegulum, **MA** = median apophysis, **p1** = process 1 of TTA, **p2** = process 2 of TTA, **vp** = ventral process of paracymbium, **dp** = dorsal process of paracymbium. Scale bars 0.5 mm.

**Figures 10–13. F4:**
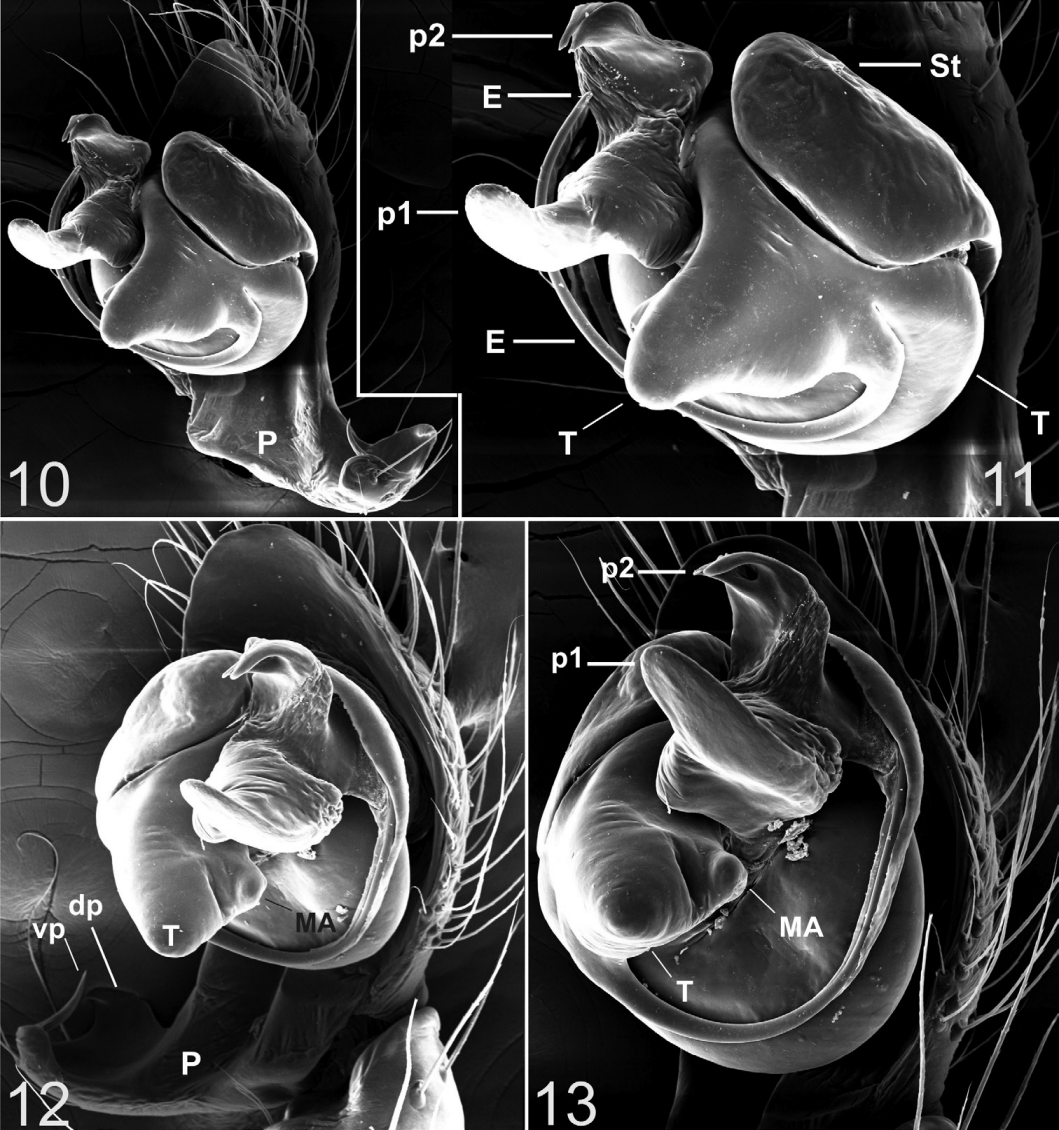
*Typhlonesticus gocmeni* sp. n. male palp. **10** nearly retrolateral view **11** ditto **12** nearly ventral view **13** ventral view. Abbreviations: **E** = embolus, **T** = tegulum, **St** = subtegulum, **MA** = median apophysis, **p1** = process 1 of TTA, **p2** = process 2 of TTA, **P** = paracymbium, **vp** = ventral process of paracymbium, **dp** = dorsal process of paracymbium.

***Measurements*** (holotype ♂): carapace length 1.15, width 0.88, opisthosoma length 1.60, width 0.84. Total length = 2.75.

**Table:** 

Leg	coxa	troc.	femur	patella	tibia	meta.	tarsus	total
**I**	0.40	0.20	3.28	0.52	3.48	3.12	1.13	12.13
**II**	0.28	0.20	2.20	0.45	2.20	2.00	0.88	8.21
**III**	0.25	0.18	1.75	0.43	1.45	1.55	0.68	6.29
**IV**	0.30	0.20	2.42	0.40	2.15	1.95	0.89	8.31

##### Female.

**Figures 14–17. F5:**
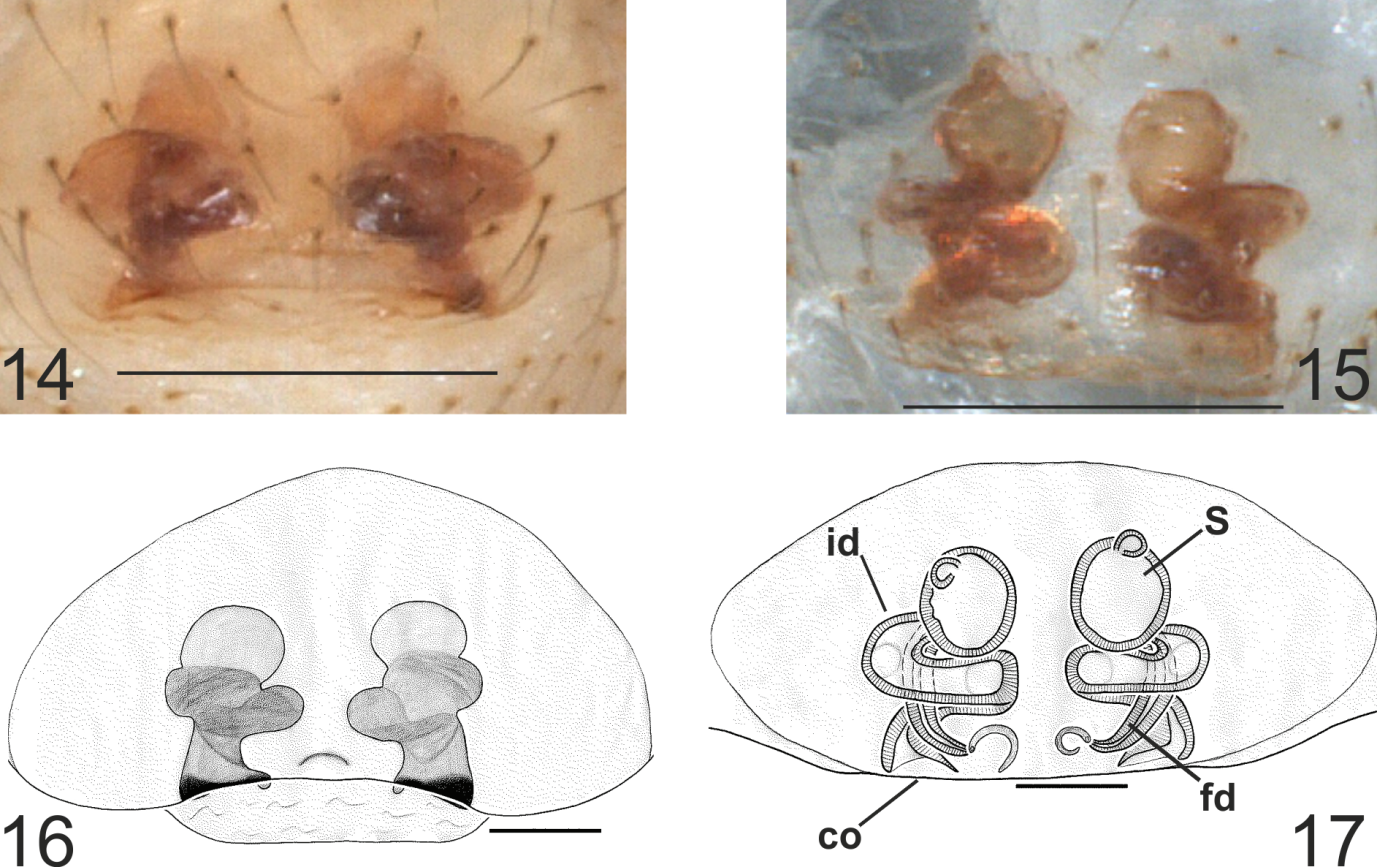
*Typhlonesticus gocmeni* sp. n. epigyne and vulva. **14** epigyne ventral view **15** vulva ventral view, **16** ditto **17** vulva dorsal view. Abbreviations: **co** = copulatory orifice, **id** = insemination duct, **fd** = fertilization duct, **S** = spermatheca. Scale bars **14–15** 0.25 mm **16–17** 0.1 mm.

All somatic characters as in male but slightly larger in size. **Epigyne** convex and prominent, without sclerotized plates (Fig. 14). The posterior edge is slightly sclerotized. Spermathecae and insemination ducts can be observed through the tegument. **Vulva** quite simple (Figs 15–17), consisting of two almost spherical spermathecae, insemination and fertilization ducts. Insemination duct coiled, forming two laps around the fertilization duct before reaching the spermatheca. Vulval pockets absent.

**Figures 18–21.**
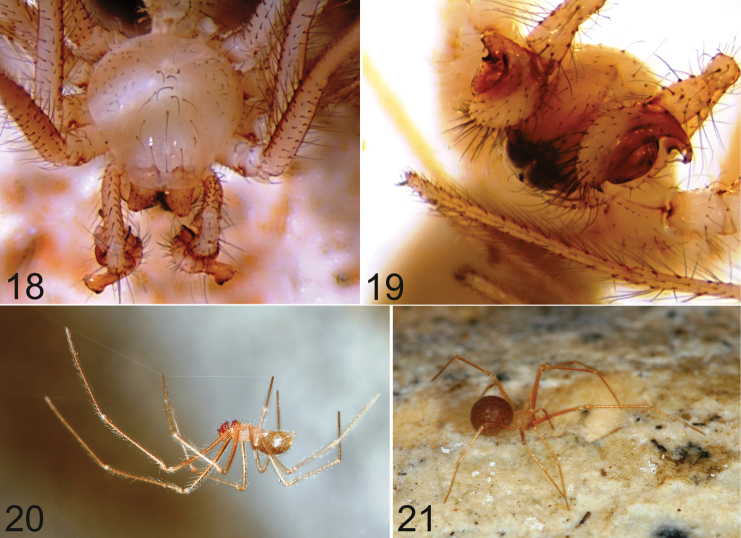
*Typhlonesticus gocmeni* sp. n. **18–19** male, prosoma **18** dorsal view **19** frontal view **20** male in the web **21** female.

***Measurements*** (paratype ♀): carapace length 1.20, width 1.00, opisthosoma length 2.04, width 1.28. Total length = 3.24.

**Table:** 

Leg	coxa	troc.	femur	patella	tibia	meta.	tarsus	total
**I**	0.38	0.15	2.64	0.45	3.40	2.80	1.11	10.93
**II**	0.32	0.13	2.48	0.45	2.25	1.95	0.93	8.51
**III**	0.20	0.13	1.75	0.34	1.40	1.20	0.61	5.63
**IV**	0.37	0.14	2.52	0.42	2.36	1.88	0.97	8.66

##### Distribution.

*Typhlonesticus gocmeni* sp. n. is only known from the type locality. This new species was previously identified as *Nesticus morisii* by Aydın Topçu and collaborators (2013). The cave is located at the northern part of the West Taurus karst region; on the east side of the Acıpayam polje, at the south eastern slope of the Karadağ hill, about 200 m above the polje level (MTA 1998). It is a fossil cave, almost horizontal and 145 m long with a roof height varying between 1–9 m with many calcite speleothem formations. It is one of the tourist caves in Turkey, open to public access since 2003, with formed tracks and fixed lighting that extend almost the full length of the cave. Specimens were collected or observed during 3 visits on 20.03.2011, 10.07.2011 and 16.10.2011. Adult specimens from both sexes were only collected in July, but also observed in October; while only subadults could be found in March. Specimens were abundant in the dark zone all along the cave, located on their webs build on the speleothem formations.

##### Molecular data.

Specimens, locality and sequences with corresponding GenBank accession numbers analyzed in the present study are listed in Appendix 1. The final concatenated dataset of the three partial genes sequences includes 13 terminals and 1807 aligned characters (*cox1* = 1049, *rrnL* = 420 and H3 = 338). Primer fidelity across taxa was not always consistent in *cox1*, consequently some specimens have slightly truncated sequence lengths. Uncorrected *cox1* genetic divergences among terminal taxa, and uncorrected genetic *cox1* divergences within and between the analyzed genera are summarized in Appendices 3 and 4.

Figure 1 shows the ML tree inferred using the combined data matrix. The new species groups with *Typhlonesticus absoloni*, *Nesticus morisii*, *Nesticus obcaecatus* and *Nesticus idriacus*. These five species constitute a highly supported evolutionary lineage (bootstrap support = 100). The remaining species included in the analysis belong to the genera *Nesticus* and *Carpathonesticus*, which constitute independent and highly supported evolutionary lineages as well. *Typhlonesticus gocmeni* sp. n. occupies a basal position in the *Typhlonesticus* clade, and is the sister species of the European representatives. Within this lineage the evolutionary relationships of the species are poorly supported (low bootstrap supports).

The mean uncorrected p-distances of *cox1* between and within taxa analyzed (Appendices 3–4) show high values. The mean p-distance between genera ranges from 11.29% (*Nesticus* versus *Carpathonesticus*) to 17.19 (*Typhlonesticus* versus *Carpathonesticus*). Also, the average evolutionary divergence within the representatives of the three Mediterranean genera analyzed ranges from 6.43% (*Carpathonesticus*) to 11.11% (*Typhlonesticus*).

## Discussion

This paper describes a new species belonging to the genus *Typhlonesticus*. The molecular phylogenetic analysis including representatives of *Nesticus*, *Carpathonesticus* and *Typhlonesticus*, all of them from the Mediterranean basin indicates that the new species lies with *Typhlonesticus absoloni* along with *Nesticus morisii*, *Nesticus obcaecatus* and *Nesticus idriacus*. These five species form a highly supported clade (bootstrap value = 100) suggesting that all of them constitute a well-defined evolutionary lineage. Accordingly, we propose the following new combinations:

*Typhlonesticus idriacus* (Roewer, 1931), comb. n., ex *Nesticus*

*Typhlonesticus morisii* (Brignoli, 1975), comb. n., ex *Nesticus*

*Typhlonesticus obcaecatus* (Simon, 1907), comb. n., ex *Nesticus*.

These new data increase significantly the distribution of the genus, which is spread throughout the northern Mediterranean, from the Iberian Peninsula to Turkey.

To date, the *Typhlonesticus* generic diagnosis has been based on a single species (Kratochvíl 1933; Deeleman-Reinhold 1974; Lehtinen and Saaristo 1980). Certainly, the five species that currently constitute this genus will allow a more comprehensive diagnosis. Unfortunately we have no males of *Typhlonesticus absoloni* nor of *Typhlonesticus morisii* (see material examined). Moreover, a molecular systematic study of all Mediterranean Nesticidae is currently being performed to test the validity of the current generic status. Therefore, we believe it appropriate to postpone an extensive generic redescription until a robust phylogenetic framework for Mediterranean nesticids has been established and until we can examine males of *Typhlonesticus absoloni* and *Typhlonesticus morisii*.

A very special trait of these species is that all of them have highly troglomorphic characters, such as the absence of eyes or reduced eye size and number (only six eyes in *Typhlonesticus obcaecatus*) and lack of body pigment. In addition, most of these species are known from a single or a small number of caves, and all of them have very narrow ranges. On other hand, the uncorrected genetic distances of *cox1* between *Typhlonesticus gocmeni* n. sp., *Typhlonesticus absoloni*, *Typhlonesticus morisii*, *Typhlonesticus obcaecatus* and *Typhlonesticus idriacus* range between 10.03 to 12.19%. Assuming an average substitution rate for arthropod mitochondrial genes of between 2% (DeSalle et al. 1987) and 2.3% (Brower 1994) we can conclude that the origin of these species preceded the Pleistocene glacial cycles. These data alongside its phylogenetic uniqueness (basal position and a deep genetic distance from the other Mediterranean genera) suggest that these species constitute an indigenous, proper fauna of Southern Europe and the Middle East and should be considered as primitive wildlife relicts, representative of a tropical or subtropical climate fauna, that should be serious candidates for protection through conservation.

## Supplementary Material

XML Treatment for Typhlonesticus gocmeni

## Appendix 1

**Table:** Species included in the phylogenetic analysis and GenBank accession numbers for the *cox1*, *rrnL* and *H3* partial sequences.

Species	Locality	cox1	rrnL	H3
*Typhlonesticus gocmeni* sp. n.	Keloğlan Cave. Dodurgalar Town, Acıpayam District. Denizli Province. Turkey, (37°23'14.74"N, 29°34'18.29"E)	KF939310	KF939307	KF939313
*Typhlonesticus absoloni* (Kratochvíl, 1933)	Baba Tusha Cv. Trnovo, Virpazar Distr., Montenegro, (N42°17'25,1'' / E019°02'10,8'' / 350m)	KF417410	KF417397	KF417416
*Typhlonesticus idriacus* (Roewer, 1931)	Grotte Pre Oreak, Nimis, Friuli. Italy	KF939312		
*Typhlonesticus morisii* (Brignoli, 1975)	Sotterranei del Forte di Vernante, Vernante, Cuneo, Italy	KF939311	KF939308	
*Typhlonesticus obcaecatus* (Simon, 1907)	Cueva del Molino de Aso, Boltaña, Prov. Huesca, Spain.	KF939309	EU746437	
*Nesticus eremita* Simon, 1879	Pishurka Cave. (=Paganetijeva Pécina), Korchula, KorchulaIsl., Croatia. (N 42°57,568; E 17°07,751 / 58m)	KF417414	KF417400	
*Nesticus speluncarum* Pavesi, 1873	Shpella e Dragoit, Gjirokastër, Albania	KF417405		KF417421
*Nesticus arenstorffi* Kulczyński, 1914	Chora Pecina Cave. Crni nugli, Dragalsko polje, Gorno krivoshije, Shelakov dol, Risan Distr. Montenegro. (N42°35'36,5'' / E018°41'41,6'' / 750m)	KF417407	KF417403	KF417422
*Nesticus henderickxi* Bosselaers, 1998	Kournas Cave. Kournas. Crete	KF417409	KF417404	
*Carpathonesticus hungaricus* (Chyzer, 1894)	Pestera Cave. Liliecilor, Cheile Ampoitei Gorges, Romania. (46°08'21.7748"N, 23°23'39.8507"E)	KF417412	KF417402	KF417419
*Carpathonesticus lotriensis* Weiss, 1983	Unnamed Cave in Lotrioara Valley, Lotrului Mountains, Sibiu, Romania. (45°34'45.8319" / 24°11'16.7493")	KF417413	KF417399	KF417418
*Carpathonesticus simoni* (Fage, 1931)	Unnamed Cave in Bisbrita Gorges, Stogu-Vinturarita Mts., Romania. (45°11'42.2789"N, 024°02'03.2702"E / 491m)	KF417408	KF417398	KF417417
*Canarionesticus quadridentatus* Wunderlich, 1992	Cv. Felipe Reventón, Icod de los Vinos, Tenerife, Canary Islands. (28°21'00.7727"N, 016°42'17.1028"W / 595m)	KF417411		KF417415

## Appendix 2

**Table:** Primers and conditions used in the present study.

Gene fragment	Primer	Or.	Sequence (5'-3')	PCR Conditions	Reference
*cox1*	C1-J-1718	F	GGAGGATTTGGAAATTGATTAGTTCC	94°(1'); 94°(30"), 45°(30"), 72°(80") × 35; 72°(4')	Simon et al. 1994
	C1-N-2191	R	CCCGGTAAAATTAAAATATAAACTTC		Simon et al. 1994
	C1-J-2183	F	CAACATTTATTTTGATTTTTTGG	94°(1'); 94°(30"), 45°(30"), 72°(80") × 35; 72°(4')	Simon et al. 1994
	C1-N-2776	R	GGATAATCAGAATATCGTCGAGG		Hedin and Maddison 2001
*rrnL*	LR-N-13398	F	CGCCTGTTTATCAAAAACAT	94°(1'); 94°(30"), 45°(35"), 72°(80") × 35; 72°(4')	Simon et al. 1994
	LR-J-12864	R	CTCCGGTTTGAACTCAGATCA		Arnedo et al. 2001
*H3*	H3a	F	ATGGCTCGTACCAAGCAGACVGC	94°(1'); 94°(30"), 48°(30"), 72°(80") × 35; 72°(4')	Colgan et al. 1998
	H3a	R	ATATCCTTRGGCATRATRGTGAC		Colgan et al. 1998

## Appendix 3

**Table:** Uncorrected genetic distances of *cox 1* gene between terminal taxa.

***Canarionesticus quadridentatus***												
***Typhlonesticus gocmeni* sp. n**	0.1874											
***Typhlonesticus obcaecatus***	0.2002	0.1003										
***Typhlonesticus obsoloni***	0.1941	0.1090	0.0947									
***Typhlonesticus morisii***	0.1953	0.1178	0.1078	0.1078								
***Typhlonesticus idriacus***	0.2021	0.1219	0.1144	0.1194	0.1205							
***Carpathonesticus simoni***	0.1798	0.1785	0.1639	0.1706	0.1898	0.1860						
***Carpathonesticus lotriensis***	0.1791	0.1687	0.1604	0.1715	0.1847	0.1845	0.0471					
***Carpathonesticus hungaricus***	0.1763	0.1530	0.1531	0.1608	0.1705	0.1822	0.0697	0.0775				
***Nesticus arenstorffi***	0.1880	0.1476	0.1458	0.1601	0.1774	0.1791	0.1096	0.1093	0.1007			
***Nesticus henderickxi***	0.1788	0.1504	0.1515	0.1548	0.1572	0.1798	0.1265	0.1202	0.1143	0.0905		
***Nesticus speluncarum***	0.1657	0.1485	0,1540	0.1582	0.1595	0.1694	0.1176	0.1075	0.1143	0.0791	0.0992	
***Nesticus eremita***	0.1723	0.1507	0.1508	0.1609	0.1700	0.1660	0.1140	0.1102	0.1105	0.0760	0.0925	0.0190

## Appendix 4

**Table:** Average evolutionary divergence between groups (below diagonal), standard error (above diagonal), and average evolutionary divergence within groups (d) and standard error (SE) of *cox 1* over sequence pairs.

*Canarionesticus*		0.0107	0.0106	0.0104	d	SE
*Typhlonesticus*	0.1958		0.0104	0.0086	0.1111	0.0065
*Carpathonesticus*	0.1784	0.1719		0.0091	0.0643	0.0083
*Nesticus*	0.1762	0.1596	0.1129		0.0768	0.0059
